# Supplementary material for: Pyruvate Dehydrogenase Kinase 1 inhibition mediated oxidative phosphorylation enhancement in cartilage promotes osteoarthritis progression
Source: BMC Musculoskelet Disord. 2023 Jul 20;24:597. doi: 10.1186/s12891-023-06585-6 (PMC10357736; doi:10.1186/s12891-023-06585-6)
Supplement: Supplementary file 1 — Additional file 1. [file 12891_2023_6585_MOESM1_ESM.zip › BP.Gsea.1653623667859/GOBP_MAINTENANCE_OF_PROTEIN_LOCALIZATION_IN_ORGANELLE.html]

Details for gene set GOBP\_MAINTENANCE\_OF\_PROTEIN\_LOCALIZATION\_IN\_ORGANELLE[GSEA]

|  || Dataset | 55457\_collapsed\_to\_symbols.cls.cls #OA\_versus\_normal.cls.cls #OA\_versus\_normal\_repos |
| Phenotype | cls.cls#OA\_versus\_normal\_repos |
| Upregulated in class | OA |
| GeneSet | GOBP\_MAINTENANCE\_OF\_PROTEIN\_LOCALIZATION\_IN\_ORGANELLE |
| Enrichment Score (ES) | 0.41932866 |
| Normalized Enrichment Score (NES) | 1.5647244 |
| Nominal p-value | 0.014669927 |
| FDR q-value | 0.28772902 |
| FWER p-Value | 1.0 |
Table: GSEA Results Summary

  

Fig 1: Enrichment plot: GOBP\_MAINTENANCE\_OF\_PROTEIN\_LOCALIZATION\_IN\_ORGANELLE      
 Profile of the Running ES Score & Positions of GeneSet Members on the Rank Ordered List

  

| SYMBOL | TITLE | RANK IN GENE LIST | RANK METRIC SCORE | RUNNING ES | CORE ENRICHMENT || 1 | GPAA1 | glycosylphosphatidylinositol anchor attachment 1 [Source:HGNC Symbol;Acc:HGNC:4446] | 44 | 1.006 | 0.0729 | Yes |
| 2 | KDELR3 | KDEL endoplasmic reticulum protein retention receptor 3 [Source:HGNC Symbol;Acc:HGNC:6306] | 77 | 0.950 | 0.1425 | Yes |
| 3 | INSIG2 | insulin induced gene 2 [Source:HGNC Symbol;Acc:HGNC:20452] | 205 | 0.814 | 0.1943 | Yes |
| 4 | ARL2BP | ADP ribosylation factor like GTPase 2 binding protein [Source:HGNC Symbol;Acc:HGNC:17146] | 208 | 0.813 | 0.2559 | Yes |
| 5 | TSPO | translocator protein [Source:HGNC Symbol;Acc:HGNC:1158] | 692 | 0.595 | 0.2635 | Yes |
| 6 | RER1 | retention in endoplasmic reticulum sorting receptor 1 [Source:HGNC Symbol;Acc:HGNC:30309] | 724 | 0.588 | 0.3057 | Yes |
| 7 | ARL2 | ADP ribosylation factor like GTPase 2 [Source:HGNC Symbol;Acc:HGNC:693] | 1179 | 0.481 | 0.3068 | Yes |
| 8 | AKT1 | AKT serine/threonine kinase 1 [Source:HGNC Symbol;Acc:HGNC:391] | 1192 | 0.478 | 0.3422 | Yes |
| 9 | BBS4 | Bardet-Biedl syndrome 4 [Source:HGNC Symbol;Acc:HGNC:969] | 1404 | 0.442 | 0.3593 | Yes |
| 10 | PARK7 | Parkinsonism associated deglycase [Source:HGNC Symbol;Acc:HGNC:16369] | 1406 | 0.442 | 0.3928 | Yes |
| 11 | KDELR2 | KDEL endoplasmic reticulum protein retention receptor 2 [Source:HGNC Symbol;Acc:HGNC:6305] | 1486 | 0.431 | 0.4193 | Yes |
| 12 | PINK1 | PTEN induced kinase 1 [Source:HGNC Symbol;Acc:HGNC:14581] | 2097 | 0.350 | 0.3984 | No |
| 13 | KDELR1 | KDEL endoplasmic reticulum protein retention receptor 1 [Source:HGNC Symbol;Acc:HGNC:6304] | 2227 | 0.336 | 0.4139 | No |
| 14 | BARD1 | BRCA1 associated RING domain 1 [Source:HGNC Symbol;Acc:HGNC:952] | 3051 | 0.245 | 0.3685 | No |
| 15 | TOPORS | "TOP1 binding arginine/serine rich protein, E3 ubiquitin ligase [Source:HGNC Symbol;Acc:HGNC:21653]" | 3244 | 0.227 | 0.3708 | No |
| 16 | CDK5 | cyclin dependent kinase 5 [Source:HGNC Symbol;Acc:HGNC:1774] | 3400 | 0.211 | 0.3747 | No |
| 17 | SYNE1 | spectrin repeat containing nuclear envelope protein 1 [Source:HGNC Symbol;Acc:HGNC:17089] | 5270 | 0.050 | 0.2332 | No |
| 18 | OS9 | OS9 endoplasmic reticulum lectin [Source:HGNC Symbol;Acc:HGNC:16994] | 5418 | 0.039 | 0.2248 | No |
| 19 | CIZ1 | CDKN1A interacting zinc finger protein 1 [Source:HGNC Symbol;Acc:HGNC:16744] | 5656 | 0.019 | 0.2078 | No |
| 20 | SP100 | SP100 nuclear antigen [Source:HGNC Symbol;Acc:HGNC:11206] | 5899 | 0.002 | 0.1891 | No |
| 21 | PML | PML nuclear body scaffold [Source:HGNC Symbol;Acc:HGNC:9113] | 6421 | -0.037 | 0.1514 | No |
| 22 | HK1 | hexokinase 1 [Source:HGNC Symbol;Acc:HGNC:4922] | 6728 | -0.058 | 0.1320 | No |
| 23 | TAPT1 | transmembrane anterior posterior transformation 1 [Source:HGNC Symbol;Acc:HGNC:26887] | 7256 | -0.100 | 0.0986 | No |
| 24 | GRIK5 | glutamate ionotropic receptor kainate type subunit 5 [Source:HGNC Symbol;Acc:HGNC:4583] | 7912 | -0.148 | 0.0589 | No |
| 25 | SKP1 | S-phase kinase associated protein 1 [Source:HGNC Symbol;Acc:HGNC:10899] | 8454 | -0.190 | 0.0313 | No |
| 26 | PDIA2 | protein disulfide isomerase family A member 2 [Source:HGNC Symbol;Acc:HGNC:14180] | 8961 | -0.229 | 0.0093 | No |
| 27 | TXN | thioredoxin [Source:HGNC Symbol;Acc:HGNC:12435] | 9487 | -0.273 | -0.0108 | No |
| 28 | HNRNPU | heterogeneous nuclear ribonucleoprotein U [Source:HGNC Symbol;Acc:HGNC:5048] | 9847 | -0.306 | -0.0155 | No |
| 29 | HK2 | hexokinase 2 [Source:HGNC Symbol;Acc:HGNC:4923] | 10030 | -0.325 | -0.0050 | No |
| 30 | MORC3 | MORC family CW-type zinc finger 3 [Source:HGNC Symbol;Acc:HGNC:23572] | 10037 | -0.325 | 0.0192 | No |
| 31 | INSIG1 | insulin induced gene 1 [Source:HGNC Symbol;Acc:HGNC:6083] | 10264 | -0.347 | 0.0280 | No |
| 32 | RANGAP1 | Ran GTPase activating protein 1 [Source:HGNC Symbol;Acc:HGNC:9854] | 10288 | -0.349 | 0.0527 | No |
| 33 | NR5A1 | nuclear receptor subfamily 5 group A member 1 [Source:HGNC Symbol;Acc:HGNC:7983] | 10308 | -0.351 | 0.0779 | No |
| 34 | SUN1 | Sad1 and UNC84 domain containing 1 [Source:HGNC Symbol;Acc:HGNC:18587] | 10348 | -0.355 | 0.1018 | No |
| 35 | SUN2 | Sad1 and UNC84 domain containing 2 [Source:HGNC Symbol;Acc:HGNC:14210] | 10366 | -0.357 | 0.1276 | No |
| 36 | HSPA5 | heat shock protein family A (Hsp70) member 5 [Source:HGNC Symbol;Acc:HGNC:5238] | 10820 | -0.407 | 0.1233 | No |
| 37 | SUPT7L | "SPT7 like, STAGA complex subunit gamma [Source:HGNC Symbol;Acc:HGNC:30632]" | 11540 | -0.506 | 0.1057 | No |
Table: GSEA details [plain text format]

  

Fig 2: GOBP\_MAINTENANCE\_OF\_PROTEIN\_LOCALIZATION\_IN\_ORGANELLE      
 Blue-Pink O' Gram in the Space of the Analyzed GeneSet

  

Fig 3: GOBP\_MAINTENANCE\_OF\_PROTEIN\_LOCALIZATION\_IN\_ORGANELLE: Random ES distribution      
 Gene set null distribution of ES for **GOBP\_MAINTENANCE\_OF\_PROTEIN\_LOCALIZATION\_IN\_ORGANELLE**
